# Comparison of chest ultrasound features to chest radiography in the diagnosis for pediatric tuberculosis: a cross-sectional study

**DOI:** 10.11604/pamj.2024.49.95.45265

**Published:** 2024-11-27

**Authors:** Geoffrey Erem, Caroline Otike, Maxwell Okuja, Faith Ameda, Dorothy Irene Nalyweyiso, Samuel Bugeza, Aloysius Gonzaga Mubuuke, Michael Kakinda

**Affiliations:** 1 Department of Radiology, School of Medicine, Makerere University, Kampala, Uganda,; 2 Department of Radiology, St. Francis Hospital, Nsambya, Kampala, Uganda,; 3 Department of Data Joint Clinical Research Center, Kampala, Uganda,; 4 Department of Radiology, Mulago National Referral Hospital, Kampala, Uganda,; 5 Department of Health Policy Planning and Management, School of Public Health, Makerere University, Kampala, Uganda

**Keywords:** Chest ultrasound, chest radiograph, comparison, screening, pediatric tuberculosis

## Abstract

**Introduction:**

robust data on the utility of chest ultrasound scans (CUS) for triage and diagnosis of pediatric tuberculosis (TB) are lacking. Therefore, we aimed to compare CUS features to chest radiography (CXR), which is the recommended imaging modality in children with presumptive pulmonary tuberculosis (PTB).

**Methods:**

eighty children ≤14 years of age with presumptive TB underwent CUS and CXR performed by two separate radiologists for each modality, who looked for the presence of consolidation, lymphadenopathy, and pleural effusion in both modalities. These were compared using Fisher’s exact test for independence to determine whether there was a significant difference in the findings between the two modalities. Cohen's kappa coefficient was used to calculate the agreement between CXR and CUS. STATA version 15 was used for analysis.

**Results:**

the proportions of children with abnormal findings (consolidation, lymphadenopathy, and pleural effusions) on CUS were 65% (52/80) and 81.3% (65/80) on CXR. Sixty-two-point-five percent (62.5% (33/52)) of those with abnormal findings on CUS and 51.3% (33/65) on CXR were likely to have TB. The overall agreement for these characteristics was moderate (κ-0.42). The differences in the findings of consolidation and pleural effusion were not statistically significant on either CXR or CUS, whereas that of lymphadenopathy was statistically significant (P<0.001).

**Conclusion:**

chest ultrasound scans detected more abnormalities in children with presumptive TB. Overall, the findings were comparable to those of CXR, except for lymphadenopathy. Ultrasound is a promising triage and diagnostic tool for pediatric TB.

## Introduction

In 2022, there were 1.3 million children estimated to have TB. However, only 46.27% (600,000) were notified to the National TB Programs (NTPs) [1]. The diagnosis of pediatric TB is missed because the signs and symptoms of pediatric TB are nonspecific and mimic those of other common childhood illnesses [2,3]. However, due to pediatric TB´s paucibacillary nature, microbiological tests to confirm the disease have low sensitivity [4]. Therefore, confirmation of pediatric TB is an exception, not the norm. Due to these challenges, clinical diagnostic algorithms with or without chest X-rays are often used to diagnose pediatric TB [5-7]. Hence, CXR is considered a key diagnostic tool for pediatric TB [8].

Perihilar or mediastinal lymphadenopathy is the most characteristic finding of pediatric TB on chest radiography (CXR) [9-11]. Chest radiography is a two-dimensional grayscale modality in which lymph nodes are difficult to discern from overlapping vascular and mediastinal structures [9]. Additionally, there is a poor intra-and inter-reader agreement for lymphadenopathy on CXR (κ = 0.00-0.40) [12,13].

Other common CXR findings described in children with PTB are consolidation and pleural effusion, but these are also observed in other respiratory infections [14]. However, the available literature reports that pleural effusions and consolidation are more common in children with microbiologically confirmed PTB [15]. Chest radiography is the imaging modality of choice for screening and triaging pulmonary TB [16]. This is despite the exposure of children to ionizing radiation and its low availability in resource-limited settings. There is also a scarcity of equipment and skilled personnel to operate and interpret the images. In contrast, CUS devices are relatively inexpensive, portable, and do not emit ionizing radiation. Occasionally, ultrasonography may not require radiological staff [17]. This makes them attractive for practical reasons in low-resource settings. In addition, CUS is a bedside test that can be performed in less than 8 minutes [14].

However, robust data regarding the use of CUS are lacking [15,17-20]. Therefore, this study aimed to compare CUS and CXR features in children with presumptive PTB. This may result in considering CUS as a modality for triaging and diagnosing pediatric TB.

## Methods

**Study design and settings:** this hospital-based cross-sectional study was conducted at the Mulago National Referral Hospital, Kampala, Uganda, from December 2020 to May 2021. On average, 12 children are diagnosed with TB monthly at this hospital [21]. These children were diagnosed based on clinical signs and symptoms, with a review of a chest radiograph if available, and GeneXpert *Mycobacterium tuberculosis/rifampicin* (MTB/RIF) ultra-testing if a sputum sample could be obtained according to national guidelines [22]. There is additional detail on the methodology in the primary article on the diagnostic accuracy of chest ultrasound scans for pediatric TB [23].

**Study participants:** the study included children aged 0-14 years. These children either had a cough of any duration with HIV or a cough of >2 weeks without HIV and with or without any of the following signs and symptoms: weight loss or failure to thrive over the last three months, persistent fever for >2 weeks that did not respond to any treatment and having been in contact with someone with confirmed PTB. Children were excluded from the study if they were already on TB treatment or TB prophylaxis for >72 hours. They were also ineligible if their parents did not consent for those ≤eight years or if they did not assent for those ≥eight years. Participants who did meet the inclusion were enrolled in the study purposively until the sample size was met from pre-selected wards.

**Radiological tests:** all study participants underwent a CUS and CXR. They were interpreted by radiologists with a range of experience from four years to 12 years. The images were interpreted by four radiologists, two for CUS and two for CXR. Each radiologist interpreted all the images independently. If there was a discrepancy, they sought consensus with the principal investigator (PI), who was also a radiologist with at least 15 years of experience acting as an arbitrator. The radiologists who interpreted the CUS were blinded to the CXR findings.

**Chest ultrasound scan:** a portable, low-cost, greyscale ultrasound machine (Edan, Model U60) with a bandwidth of 5.0 to 10.0 MHz linear probes was used to scan the chest of the children in this study. The radiologists were responsible for methodological scanning of the children while looking for consolidations, lymph nodes, and pleural effusions. The older children were scanned either sitting or supine plus in left and right lateral decubitus positions to examine the lungs and pleura, while the younger children were scanned on the laps of the caregiver. The chest was divided into 14 regions: the left and right, anterior, lateral, posterior, superior, and inferior regions, and the two apices. All regions were scanned in longitudinal and transverse (intercostal) planes. Mediastinal ultrasound was performed through the suprasternal notch. The child was placed in the supine position with the neck slightly extended to improve access to the suprasternal notch, and transverse and oblique views were obtained. All the cine clips were saved.

**Chest radiograph:** the CXR images (the posterior-anterior and lateral) were independently reviewed by two radiologists who reported on a standardized record sheet for pleural effusions, lymphadenopathy, and consolidations, along with the radiological diagnosis of the likelihood of TB. In cases of disagreement on the final diagnosis, a third radiologist was consulted, and the final diagnosis was based on a consensus opinion. All the images were digitally achieved.

**Outcome measures:** the outcome measures are TB likely or TB unlikely on either CXR or CUS. Where TB likely is the presence of signs and symptoms suggestive of TB plus or minus contact with someone with TB and CXR or CUS suggestive of TB. While TB is unlikely, there is the presence of signs and symptoms of TB plus or minus contact with someone with TB, but CXR or CUS are not suggestive of TB.

**Data analysis:** the study characteristics were summarized using mean and standard deviation (SD) for normally distributed numerical data and median with interquartile range (IQR) for non-normally distributed numerical data. Categorical data are summarized using frequencies and proportions. The presence of consolidation, lymph nodes, and pleural effusion on both modalities (CXR and CUS) was compared using Fisher´s exact test for independence to determine if there was a significant difference in the findings between the two tests. Cohen's kappa coefficient was used to calculate the agreement between CXR and CUS. The following interpretations of the results were used: less than zero, no agreement; 0 to 0.20 slight, 0.21 to 0.40 fair, 0.41 to 0.60 moderate, 0.61 to 0.80, substantial and 0.81 to 1, almost perfect agreement. STATA version 15 was used for analysis.

**Ethical approval and consent to participate:** ethical approval to conduct this study was granted by the School of Medicine Research Ethics Committee at Makerere University (REC No. 2020-138). The investigations used in the study were part of the standard of care for a long time for different purposes but not for the diagnosis of PTB. Informed parental or caregiver consent was obtained for children ≤8 years of age, while assent was obtained from children >8 years of age, which is a requirement in Uganda. However, all methods were performed per the relevant guidelines and regulations or declaration of Helsinki.

## Results

**Participants’ characteristics:** of the eighty study participants, 42 (52.5%) were male, with an average age of 42.9 months (range 1-144). Most participants (40/80, 50.0%) were between 1-55 years old, while 17 (12,80, 15.0%) and 11 (13.7%) were aged 5-10 and 10-15 years respectively, the rest were below one year (Table 1).

**Table 1:** characteristics of the study participants

Characteristic	Total participants (n=80)
**Sex, n (%)**	
Male	42 (52.5)
Female	38 (47.5)
**Mean age in months**	42.89
**Age ranges, n (%)**	
< 1 year	17 (21.3)
1-<5 years	40 (50.0)
5 years -<10 years	12 (15.0)
10 years-<15 years	11 (13.7)

**Comparison of chest ultrasound findings and chest radiography findings:** a total of 65% of the children had abnormal findings on CUS, while 81.3% had abnormal CXR findings. Among those with abnormal findings, 62.5% were likely to have TB on CUS and 51.3% were likely to have TB on CXR. Among the 80 children, 38.8% showed consolidation on CUS, and 93.5% of these while on CXR, 27 children (33.75%) showed consolidation, and all were likely to have TB. There was no significant difference in consolidation findings between CUS and CXR (P=0.491). In 20.0% of CUS scans, pleural effusion was detected, with all cases likely to have TB. 13.8% of CXRs showed pleural effusions, and in 90.9% of these, there was a likelihood of TB. There was no significant difference in pleural effusion findings between CUS and CXR (P=0.407)). Among the 5 children with lymphadenopathy on CUS, all were likely to have TB. In contrast, only 4 of 27 children with lymphadenopathy on CXR were likely to have TB. The differences in lymphadenopathy findings between CUS and CXR were significant (P<0.001) (Table 2).

**Table 2:** comparing chest ultrasound scan and chest radiography features

	CXR findings		CUS findings		
Features	TB likely, n (%)	TB unlikely, n (%)	TB likely, n (%)	TB unlikely, n (%)	Fisher's exact test (p-value)
**Consolidation**					0.491
Yes	27 (81.8)	0 (0.0)	29 (59.2)	2 (6.5)	
No	6 (18.2)	47 (100.0)	20 (40.8)	29 (93.5)	
**Pleural effusion**					0.407
Yes	10 (30.3)	1 (2.1)	16 (32.7)	0 (0.0)	
No	23 (69.7)	46 (97.9)	33 (67.3)	31 (100.0)	
**Lymph adenopathy**					
Yes	4 (80.00)	23 (30.77)	5 (10.2)	0 (0.0)	<0.001
No	1 (20.00)	52 (69.3)	44 (89.8)	31 (100.0)	

Where n= is the number of children with or without a particular feature; CXR: chest radiography; CUS: chest ultrasound scans; TB: tuberculosis

**Inter-reader agreement on chest ultrasound scan and chest radiograph:** Table 3 shows the agreement between CUS and CXR. The agreement for consolidation and pleural effusion on the CUS and CXR was moderate (κ-0.41 and κ-0.43, respectively), while that for lymphadenopathy was slight (κ-0.16). The overall agreement for the likelihood of TB was moderate (κ-0.42) for both CUS and CXR.

**Table 3:** inter-reader agreement of chest ultrasound scan and chest radiography

Imaging finding	Proportions on chest X-ray	Proportions on chest ultrasound scans	Degree of agreement	Kappa kohen statistics
Consolidation	27/80	31/80	58/80	0.407
Pleural effusion	11/80	16/80	67/80	0.425
Lymph adenopathy	27/80	5/80	56/80	0.162
Overall (tuberculosis likely)	33/80	49/80	56/80	0.423

## Discussion

We set out to compare the following features (consolidation, pleural effusion, and lymphadenopathy) on CUS and CXR in children with presumptive TB. There was no significant difference in finding consolidations and pleural effusions between CUS and CXR, unlike lymphadenopathy. When the reading of these features was compared between CUS and CXR, there was a moderate agreement for consolidations and pleural effusions, but only a slight agreement for lymphadenopathy.

Slightly more pleural effusions were detected on CUS (20%) than on CXR (13.75%), but the difference was not statistically significant (P=0.407). This is comparable to the literature that reported CUS to be superior in detecting pleural effusion than CXR [14,24,25]. Pleural effusion is more likely to be associated with pulmonary TB on both CXR and CUS, although pleural effusion can occur in children with pneumonia caused by other bacteria (pneumococcus and Staphylococcus) or viruses [14]. However, these children are usually younger and acutely unwell compared with children with pleural effusion due to TB [26]. Pulmonary tuberculosis is a probable diagnosis when pleural effusion is present, especially if there is consolidation.

The presence of consolidation was associated with the likelihood of TB on both the CUS and CXR. Consolidation is commonly seen in children with pulmonary TB. However, it has also been observed in children with bacterial infections and, less commonly, viral pneumonia. “Co-infection” was also common, where children with underlying pulmonary TB present co-infected with viral or bacterial pneumonia [26]. We encourage radiologists and clinicians in the presence of consolidation to look for other features, especially pleural effusion. Less lymphadenopathy was detected on CUS than on CXR. This contrasts with the available literature, which reported more lymphadenopathy in CUS than in CXR [14,18]. Lymphadenopathy with or without parenchymal abnormalities is the radiological hallmark of primary TB in children. This is often more prevalent in children aged <3 years than in those aged 4 and 15 years [27]. Since lymphadenopathy, especially in the mediastinal region, is likely to be the only feature on CXR, CUS efforts should be made to look for it in presumptive TB patients, especially those less than 3 years of age.

This study has key implications for clinical practice, especially in the management of children with suspected TB, using ultrasonography as a screening tool. Chest ultrasound scans can detect some features suggestive of TB in children and have the potential to be used as an initial modality before children are exposed to chest X-rays with ionizing radiation. Chest ultrasound scans could become more useful in rural and remote healthcare settings especially if accompanied by prerequisite capacity building and the technology becomes more accessible and affordable Thus, we encourage the screening of suspected children in these settings initially with CUS where available and the prerequisite skills and knowledge to use it are available.

This study compared CUS and CXR findings. Ideally, the finding should have been compared to a reference standard. There are a couple of options that can be considered. These are referencing confirmation of disease by either using culture or MTB GeneXpert Ultra. However, these have a low sensitivity so many patients with TB will be missed. The other would be using response to treatment however this is very susceptible to loss of follow-up [28]. We could also have considered comparing CUS with computed tomography (CT) and Magnetic Resonance and Imaging (MRI), which are the reference standards for imaging. However, a Chest CT scan has a very high radiation dose, making it not a first-line imaging modality for children with radiosensitive tissues [29]. Magnetic resonance imaging scans are few and very expensive in the setting in which the study was conducted. Despite this, however, the study does provide useful indicators that CUS can be used as an initial screening tool for children suspected of having TB and aids in identifying those that require further imaging and management. There is a need to compare the additional benefits of using CUS to diagnose TB in children earlier.

This study had several other limitations. The consensus case definition of childhood TB was not fully considered [30]. Despite the use of signs and symptoms suggestive a CXR was consistent with tuberculosis. Other criteria, such as a positive response to treatment and immunologic evidence of *M. tuberculosis* infection was not considered for likely or unconfirmed TB. In addition, the radiologists subjectively read both CXR and CUS, but this is likely to have been countered by their level of experience.

Our study compares the findings of CUS with those of CXR. Although not new, it does add to the body of evidence showing that it is feasible to replace CXR in resource-limited settings. This complements the work of Heuvelings *et al*. [14]. However, our study was from a radiologist´s point of view and did not seek to compare the findings between the two imaging modalities, whether TB was unlikely, unconfirmed, or confirmed.

## Conclusion

Chest ultrasound scans detected more abnormalities in children who were likely to have TB. Its findings were comparable to those of CXR in finding features other than lymphadenopathy. Ultrasound is a promising triage and diagnostic tool for pediatric TB. However, there is a need to compare the CUS with the reference standard.

### What is known about this topic

- Chest radiograph is the imaging modality of choice for diagnosis of pediatric TB;
- Chest radiograph uses ionizing radiation which changes the nature or destroys the deoxyribonucleic acid (DNA);
- A chest ultrasound scan can be performed in under 9 minutes.

### What this study adds

- Chest ultrasound scan can detect more features than chest radiography of presumptive TB;
- Chest ultrasound scan can be used in place of chest radiography for triage and diagnosis of pediatric TB.
